# Elucidating the Importance of DOT1L Recruitment in MLL-AF9 Leukemia and Hematopoiesis

**DOI:** 10.3390/cancers13040642

**Published:** 2021-02-05

**Authors:** Sierrah M. Grigsby, Ann Friedman, Jennifer Chase, Bridget Waas, James Ropa, Justin Serio, Chenxi Shen, Andrew G. Muntean, Ivan Maillard, Zaneta Nikolovska-Coleska

**Affiliations:** 1 Molecular and Celular Graduate Program, Department of Pathology, University of Michigan Medical School, Ann Arbor, MI 48104, USA; Sierrah.Grigsby@downstate.edu (S.M.G.); jropa@umich.edu (J.R.); jtserio9@gmail.com (J.S.); chenxi.shen@gmail.com (C.S.); andrewmu@med.umich.edu (A.G.M.); 2 Department of Internal Medicine, Life Sciences Institute, University of Michigan Medical School, Ann Arbor, MI 48104, USA; friedmna@umich.edu (A.F.); jennifer.a.chase@gmail.com (J.C.); bwaas@umich.edu (B.W.); imaillar@pennmedicine.upenn.edu (I.M.); 3 Rogel Cancer Center, Michigan Medicine, University of Michigan Medical School, Ann Arbor, MI 48104, USA

**Keywords:** Dot1l, MLL-rearrangement leukemia, protein-protein interaction, hematopoiesis

## Abstract

**Simple Summary:**

MLL-rearranged leukemia, driven by MLL-fusion proteins, is an aggressive, therapy-resistant leukemia found in >60% of infant leukemia and ~10% of adult leukemia. Studies have shown that inhibiting DOT1L enzymatic activity blocks leukemogenesis. However, DOT1L is critical for various normal cellular functions, including hematopoiesis. This study aimed to show that targeting the interaction between the MLL-AF9 fusion and DOT1L would inhibit leukemogenesis while sparing non-leukemic hematopoiesis. We found that disrupting the AF9-DOT1L interaction with a single point mutation was sufficient to impair leukemogenesis. We also demonstrate that genetic interventions that result in loss of DOT1L enzymatic activity in non-leukemic cells rapidly depletes hematopoietic stem and progenitor cells within 7–10 days; however, hematopoiesis was preserved when the AF9-DOT1L interaction was disrupted, leaving the enzymatic function intact. These studies are a proof of concept demonstrating the potential therapeutic advantage of inhibiting the AF9-DOT1L interaction and disrupting the integrity of the MLL-fusion complex.

**Abstract:**

*MLL1 (KMT2a)* gene rearrangements underlie the pathogenesis of aggressive MLL-driven acute leukemia. AF9, one of the most common MLL-fusion partners, recruits the histone H3K79 methyltransferase DOT1L to MLL target genes, constitutively activating transcription of pro-leukemic targets. DOT1L has emerged as a therapeutic target in patients with MLL-driven leukemia. However, global DOT1L enzymatic inhibition may lead to off-target toxicities in non-leukemic cells that could decrease the therapeutic index of DOT1L inhibitors. To bypass this problem, we developed a novel approach targeting specific protein-protein interactions (PPIs) that mediate DOT1L recruitment to MLL target genes, and compared the effects of enzymatic and PPIs inhibition on leukemic and non-leukemic hematopoiesis. MLL-AF9 cell lines were engineered to carry mutant *DOT1L* constructs with a defective AF9 interaction site or lacking enzymatic activity. In cell lines expressing a *DOT1L* mutant with defective AF9 binding, we observed complete disruption of DOT1L recruitment to critical target genes and inhibition of leukemic cell growth. To evaluate the overall impact of DOT1L loss in non-leukemic hematopoiesis, we first assessed the impact of acute *Dot1l* inactivation in adult mouse bone marrow. We observed a rapid reduction in myeloid progenitor cell numbers within 7 days, followed by a loss of long-term hematopoietic stem cells. Furthermore, WT and PPI-deficient DOT1L mutants but not an enzymatically inactive DOT1L mutant were able to rescue sustained hematopoiesis. These data show that the AF9-DOT1L interaction is dispensable in non-leukemic hematopoiesis. Our findings support targeting of the MLL-AF9–DOT1L interaction as a promising therapeutic strategy that is selectively toxic to MLL-driven leukemic cells.

## 1. Introduction

The *Mixed Lineage Leukemia 1 (MLL1; KMT2a)* gene is located on chromosome 11q23 and encodes a histone 3 lysine 4 (H3K4) methyltransferase. MLL1 mediated H3K4me is associated with active gene transcription and is required for the regulation of a variety of targets in embryogenesis and normal hematopoiesis, including *Homeobox* (*Hox*) genes [1,2]. The *MLL1* locus contains a breakpoint region upstream of sequences encoding the SET domain that is involved in chromosomal translocations, generating oncogenic fusion proteins that combine the DNA binding N-terminus of MLL with one of over 80 different partners reported to date [3]. Some of the most common fusion partners are AF9, ENL, AF4, and AF10, which account for >90% and 50% of all MLL-driven acute lymphoblastic and myeloid leukemias, respectively [4]. Leukemias driven by MLL-fusion proteins account for approximately 10% of adult leukemias and 60% of infant leukemias, and are associated with a poor prognosis [5,6,7,8]. MLL fusion partners interact with the histone methyltransferase Disrupter of Telomeric silencing 1-Like (DOT1L) directly or indirectly, found in macromolecular complexes that promote transcriptional elongation, including the ENL-associated protein complex (EAP, containing AF4, AF5, AF9, AF10, ENL, DOT1L, and pTEFb); the AEP complex (containing AF4, AF5, ENL, and pTEFb); the super elongation complex (SEC, containing AF4, AF9, ENL, AFF4, ELL1, and pTEFb), and DotCom (DOT1-L complex, containing DOT1L, AF9, ENL, AF10, AF17 along with several WNT pathway modifiers) [9,10,11,12,13]. Despite certain variations in these reported multi-protein complexes, they collectively provide evidence that MLL fusion proteins recruit the histone methyltransferase DOT1L to MLL target genes, and that this recruitment is required for leukemogenesis. DOT1L is the only methyltransferase known to catalyze the mono-, di- and trimethylation of histone H3 at lysine 79 (H3K79) in a non-processive manner using S-adenosylmethionine as a cofactor. The presence of H3K79 methylation is highly correlated with active gene transcription [14]. DOT1L recruitment by MLL fusion proteins leads to deregulated methylation of H3K79 at *MLL* target genes and enhanced expression of a characteristic set of genes that drive leukemogenesis, including *HOXA9* and *MEIS1* [15,16,17]. Genome-wide analysis showed a distinct pattern of H3K79 methylation in human MLL-rearranged primary leukemia samples compared with normal cells as well as in selected leukemias with other cytogenetic abnormalities [18]. Inhibiting the enzymatic activity of DOT1L by genetic ablation or small-molecule inhibitors was sufficient to suppress leukemogenesis in preclinical models of MLL fusion leukemia, demonstrating a critical role for DOT1L in leukemia pathogenesis and validating DOT1L as a promising therapeutic target [15,19,20,21]. Pinometastat (EPZ-5676) is a highly potent and selective DOT1L inhibitor, targeting its catalytic domain, with on-target cellular and in vivo activity [22,23,24,25]. These promising results led to the initiation of phase 1 clinical trials [26,27].

Accumulating evidence indicates that epigenetic regulators are critical for the regulation of cellular and transcriptional homeostasis [28,29,30,31]. DOT1L’s highly conserved enzymatic activity regulates diverse cellular functions ranging from DNA repair, cell cycle regulation, cardiac function, embryogenesis, and hematopoiesis [12,32,33,34,35,36,37]. Given that DOT1L is essential both in development and normal hematopoiesis and given that a significant percent of MLL-rearranged leukemia patients are infants, it is important to develop an alternative, safer therapeutic strategy that does not inhibit key physiological functions of DOT1L.

**Figure 1:**
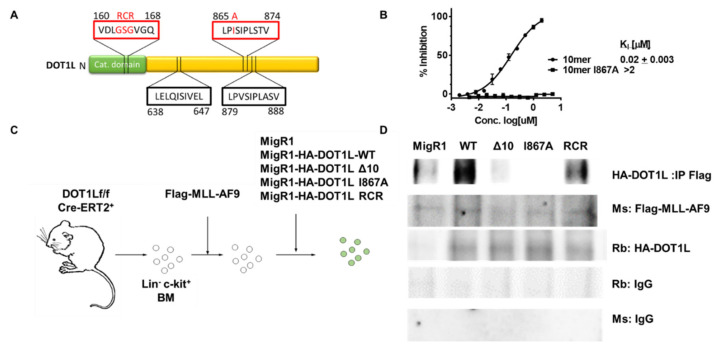
A single amino acid change blocks AF9-DOT1L protein-protein interaction. (**A**) Protein diagram of DOT1L highlighting the catalytic domain mutation from residues 163–165 and AF9 binding domains at residues 638–647, 865–874 and 879–888. The red boxes indicate regions of the protein that were mutated for the following studies. (**B**) Fluorescence Polarization assay showing the that the 10mer peptide from residues 865–874 can compete for binding with AF9 with a fluorescein-labeled DOT1L peptide with a K_I_ of 20 nM; whereas, the peptide with the I867A mutation did not show binding up to 10 µM. (**C**) Schematic of the process of generating *DOT1L f/f MLL-AF9* cell lines with different DOT1L constructs reintroduced. Cells were harvested from the bone marrow of *DOT1L f/f CreERT2* mice. The bone marrow was lineage depleted and transduced will MLL-AF9. MLL-AF9 were selected by neomycin treatment then subjected to a secondary transduction with either MigR1 as an empty vector control of one of four DOT1L constructs including, DOT1l-WT, DOT1L-Δ10, DOT1L-I867A (Δ10 and I867A mutants block AF9-DOT1L interaction), and DOT1L-RCR (enzymatically inactive DOT1L protein). These cells were then GFP sorted and used for DOT1L excision studies. (**D**) co-Immunoprecipitation (co-IP) using Flag-MLL-AF9 to pulldown HA-DOT1L in the five established murine cell lines showing disruption of the MLL-AF9 and DOT1L protein-protein interaction in the Δ10 and I867A mutants cell lines as well as the MigR1 cell line where HA-DOT1L is not present with corresponding Rb (rabbit) and Ms (mouse) IgG controls.

We previously reported biochemical, biophysical, and functional characterization of the protein-protein interactions (PPIs) between DOT1L and MLL fusions AF9/ENL. The DOT1L site that interacts with the ANC Homology Domain (AHD) domain of AF9 and ENL was mapped to a 10-amino acid region (amino acids 865–874), highly conserved in DOT1L from a variety of species. Alanine scanning mutagenesis identified DOT1L amino acids Ile 867 and Ile 869 as essential for the interaction between AF9/ENL and DOT1L. Importantly, functional studies showed that the mapped AF9/ENL interacting site is essential for immortalization by MLL-AF9, as DOT1L lacking the ten AF9 interacting residues (Δ10) showed a significant reduction in colony formation similar to the effects of DOT1L enzymatic inactivation [38]. Consistent with our findings, a recent study reported that DOT1L has three AF9 binding sites: 638–647 (site 1) and 879–888 (site 3) in addition to 865–874 (site 2) identified in our study (Figure 1A) [39]. Interestingly, the binding motifs at site 2 and site 3 have nearly identical sequences and represent a high-affinity binding motif in DOT1L. This study further confirmed that DOT1L recruitment by AF9 is necessary for MLL-AF9 mediated colony-formation and leukemic transformation. Recently we reported a class of peptidomimetics based on the 10-mer DOT1L binding peptide, providing a proof-of-concept for the development of nonpeptidic compounds to inhibit DOT1L activity by targeting its interaction with MLL-oncofusion proteins, AF9 and ENL [40].

In this study, we investigate the importance of DOT1L recruitment by MLL-AF9 in leukemic transformation as well as wild type AF9 in non-leukemic hematopoiesis. Using a genetic approach, we compared the inhibitory effects of disrupting the AF9-DOT1L interaction or DOT1L enzymatic activity in leukemia cells harboring the MLL-AF9 oncoprotein as well as in non-leukemic adult bone marrow. We show that the interaction between AF9 and DOT1L is critical for MLL-AF9 driven leukemia and blocking this PPI inhibits H3K79 methylation at MLL-target genes. This leads to downregulation of MLL-target genes, and differentiation, similar to DOT1L enzymatic inhibition. Importantly, loss of DOT1L enzymatic activity led to the rapid depletion of hematopoietic progenitor cells, followed by loss of hematopoietic stem cells and hematopoietic failure. In contrast, disrupting the AF9-DOT1L PPI allowed for sustained hematopoiesis. Overall, this study provides evidence that targeting DOT1L recruitment by the AF9 onco-fusion protein without altering enzymatic activity provides decreased hematopoietic side effects and a therapeutic advantage.

## 2. Results

### 2.1. Characterization of MLL-AF9-Transformed Cells in the Presence of Different DOT1L Constructs

Our previous work identified a direct interaction between the ANC1 homology domain (AHD) of AF9 and ENL with the DOT1L 10 amino acid region, aa 865–874 (Figure 1A) [38]. Using alanine scanning mutagenesis, we demonstrated that mutating four critical hydrophobic residues, L865, I867, I869, and L871, led to a significant >150-fold decrease in binding affinity to both fusion proteins, MLL-AF9 and MLL-ENL [41]. Using a fluorescence polarization (FP)-based assay, we confirmed that the DOT1L WT 10mer (865–874) peptide binds AF9 with a K_I_ of 20 nM. In contrast, the mutant I867A 10mer peptide did not show binding up to 10 µM (Figure 1B), demonstrating that the I867A point mutation is sufficient to disrupt the AF9-DOT1L interaction. An independent study confirmed the 10 amino-acid interaction site and identified two additional DOT1L motifs as AF9 binding sites: aa 638–647 and aa 879–888 (Figure 1A) [39].

To further evaluate the role of DOT1L as well as to explore the consequences of blocking DOT1L binding to MLL-AF9 and its recruitment to MLL target genes, we established several MLL-AF9-transformed leukemia cell lines. In these experiments, MSCV-based vectors were used to transduce mouse bone marrow cells from floxed Dot1l mice with the leukemogenic MLL-AF9 fusion protein, as described previously [35]. Several Dot1lf/f CreER(T2)^+^ cell lines were established carrying retroviral vectors expressing HA-tagged wild type human DOT1L (WT-DOT1L); DOT1L lacking the 10 amino acid AF9 binding site aa865–874 (Δ10-DOT1L); DOT1L carrying a point mutation at one of the essential hydrophobic residue, Ile867 to Ala, which completely abrogates binding of DOT1L to AF9 (I867A-DOT1L); methyltransferase inactive DOT1L where GSG is mutated into RCR in the S-adenosylmethionine-binding domain (RCR-DOT1L); and MigR1 empty vector control (Figure 1C) [19,42,43]. The expression of exogenous MLL-AF9 and that of all DOT1L constructs was validated by qRT-PCR, showing that all cell lines generated contained transcripts for both MLL-AF9 and DOT1L except the empty vector cell line expressing only MLL-AF9 (Figure S1A). This expression profile was confirmed by Western blot detection of HA-tagged DOT1L and Flag-MLL-AF9 proteins, as expected (Figure S1B).

To validate the disruption of DOT1L recruitment in Δ10-DOT1L and I867A DOT1L mutant cell lines, a co-immunoprecipitation (co-IP) was used in comparison to the negative control MigR1 cell line (expressing only GFP), as well as WT-DOT1L and RCR-DOT1L cell lines expressing DOT1L with an intact AF9-binding site. As expected, antibodies against MLL-AF9 were unable to co-immunoprecipitate the Δ10 DOT1L and I867A DOT1L proteins, whereas WT DOT1L and RCR DOT1L proteins with an intact AF9-DOT1L interaction site had preserved interactions with MLL-AF9 (Figure 1D). To further verify the interactions between AF9 and DOT1L in cells, a Duolink Proximity Ligation Assay (PLA) was used with oligonucleotide PLA probes, coupled to secondary antibodies that recognize the primary antibodies used to detect the HA (DOT1L constructs) and Flag tag (MLL-AF9). As expected, we detected interactions between MLL-AF9 and WT DOT1L or RCR DOT1L (<40 nm apart), consistent with the fact that both of these DOT1L constructs have an intact AF9 binding site and can interact with the AF9 onco-fusion protein. In contrast, the PLA signal was not observed in the Δ10-DOT1L and I867A-DOT1L mutant cell lines where DOT1L recruitment by MLL-AF9 is disrupted (Figure S1C). Overall, these results demonstrate that established MLL-AF9 transformed cell lines with different exogenous DOT1L constructs can be used to study and compare the biological effects of DOT1L mutants that block its recruitment and histone methyltransferase enzymatic activity in the context of MLL-AF9-mediated leukemic transformation.

### 2.2. Targeted Disruption of Protein-Protein Interactions between MLL-AF9 and DOT1L Suppresses Leukemia Cell Growth and Promotes Their Differentiation

**Figure 2:**
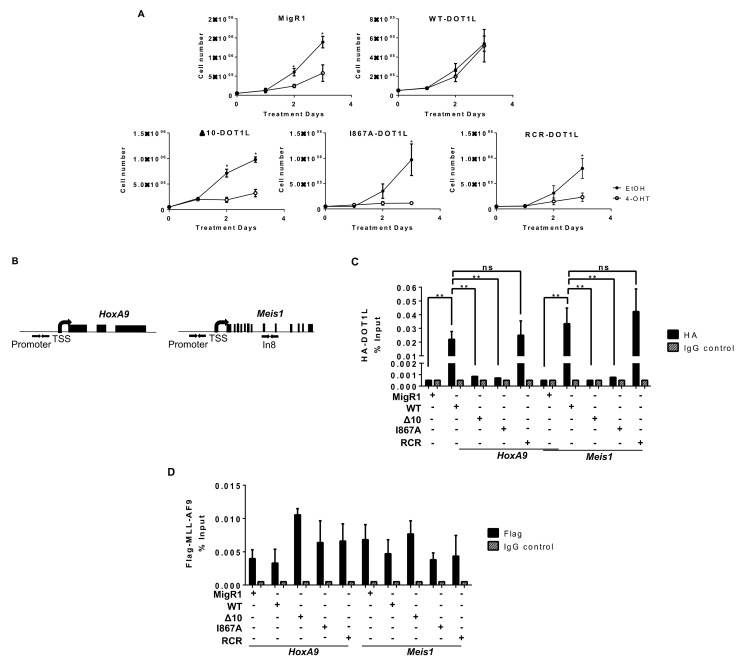
MLL-AF9 mediated proliferation is dependent on the interaction and recruitment of DOT1L. (**A**) Cells were treated at day 0 and day 2 with 7.5 nM tamoxifen (4-OHT) and monitored for cell proliferation. Mutating the AF9-DOT1L interaction site (Δ10 and I867A) significantly reduces cellular proliferation at a similar level to deletion (MigR1) or enzymatic inhibition (RCR) of DOT1L in MLL-AF9 cells (n ≥ 4). (**B**) Schematic representation of HoxA9 and Meis1 gene loci with arrows indicating the location of Chromatin Immunoprecipitation qPCR (ChIP-qPCR) primers for the promoter region. (**C**) Using an anti-HA antibody, DOT1L constructs were immunoprecipitated, showing disruption of the MLL-AF9 and DOT1L interaction at the HoxA9 and Meis1 promoter. Only WT and RCR constructs with the intact PPI interaction site were detected. There was minimal to no enrichment over background for I867A and Δ10 (representative data from two independent experiments with technical triplicates). (**D**) An anti-Flag ChIP-qPCR for MLL-AF9 showed no significant difference in localization to both HoxA9 and Meis1 promoters in each cell line with corresponding % input control (representative data from two independent experiments with technical triplicates). Significance key calculated using a *t*-test on Prizm 6 software (ns *p* > 0.05, * *p* ≤ 0.05, ** *p* ≤ 0.01).

To examine the importance of DOT1L recruitment by MLL-AF9 in leukemia cell proliferation, Dot1lf/f CreER-T2^+^ MLL-AF9 cell lines expressing wild-type DOT1L and DOT1L mutants (Δ10 deletion, I867A, and RCR) were grown in IL-3-conditioned media in the presence of 4-OHT to inactivate endogenous Dot1l. This strategy allowed us to compare the effects of blocking the interactions between the fusion oncogene protein AF9 and DOT1L with the hallmark phenotypic response to DOT1L enzymatic inhibition. Dot1lf/f CreER-T2^+^ MLL-AF9 cell lines were subjected to treatment with 4-OHT on day 0 and day 2. Dot1l excision was maintained, as shown by genotyping the cell lines at the termination of the experiment (Figure S2A). To confirm that growth defects were not due to 4-OHT toxicity or Cre recombinase activity, Dot1l^+/+^ CreER-T2^+^ murine cells were treated with 4-OHT, showing no effect on cell proliferation or on the abundance of H3K79me2 marks as assessed by western blot (Figure S3A,B). As expected, upon treatment with 4-OHT and loss of the endogenous DOT1L, cell lines carrying an empty vector or the enzymatically deficient RCR-DOT1L did not sustain cellular proliferation [15,16,38,39]. Control leukemia cells expressing WT-DOT1L showed rescue of proliferation, confirming that the observed phenotype was due to loss of DOT1L and its histone methyltransferase activity. Strikingly, bone marrow cells transduced with the MLL-AF9 fusion protein exhibited high sensitivity to blocking the protein-protein interactions between AF9 and DOT1L. Both cell lines, the Δ10-DOT1L and I867A-DOT1L, showed a significant decrease in cell proliferation to the same extent as MigR1 and RCR-DOT1L cells (Figure 2A). These findings provide strong evidence that disrupting the interactions between MLL-AF9 and DOT1L by deleting the previously identified minimum targeting module (Δ10; 865–874aa), and via a single point mutation (I867A) effectively inhibits the growth of MLL-AF9-driven leukemia cells.

**Figure 3:**
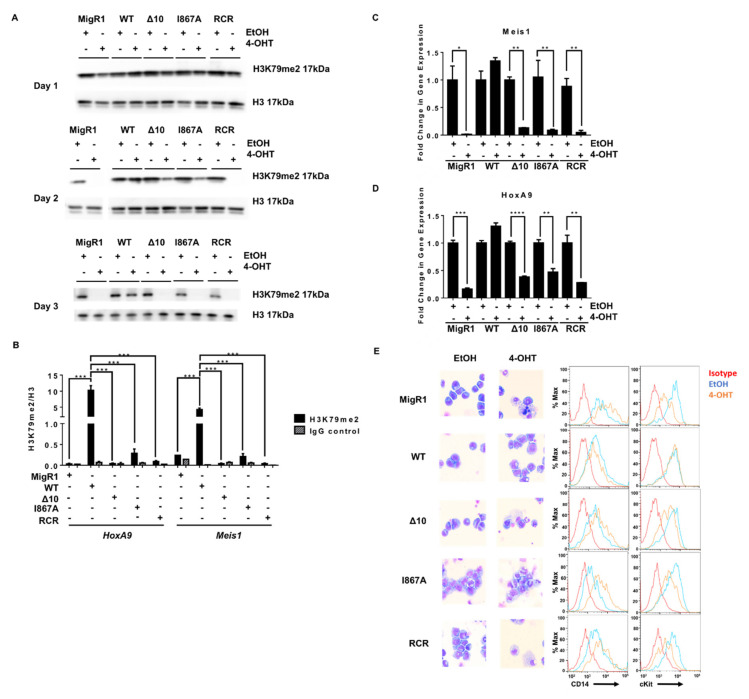
Disrupting the MLL-AF9 and DOT1L interaction has the same consequences on leukemogenesis as enzymatic inactivation of DOT1L. (**A**) H3K79me2 for Days 1–3 of the proliferation assay showing a slower decrease in protein-protein inhibition cell lines than enzymatically inactivated or empty vector controls (representative data from two or more independent experiments). (**B**) ChIP-qPCR for H3K79me2 at the HoxA9 and Meis1 promoters plotted relative to H3 for each cell line confirmed the decrease in H3K79me2 in the MigR1, Δ 10, I867A and RCR cells in comparison to WT (representative data from two independent experiments with technical triplicates). (**C**) The abundance of MLL target genes Meis1 and (**D**) HoxA9 mRNA is significantly reduced in 4-OHT-treated cells in comparison to EtOH-treated controls in the MigR1, Δ10, I867A, and RCR-transduced cells. Expression of these genes was not decreased in the WT control cell line when comparing treated and non-treated cells (n = 3). (**E**) Wright-Giemsa stain of cells after 4-OHT treatment Wright-Giemsa stain of cells after 4-OHT treatment (Olympus IX83 Inverted Microscope; Original magnification ×400) and corresponding flow cytometry for cell differentiation marker CD14 and progenitor marker c-Kit, showing cell differentiation in MigR1, Δ10, I867A, and RCR cells while WT cells retain their blast-like morphology and cell surface markers (representative data from four independent experiments).Significance key calculated using a *t*-test in Prism 6 software (ns *p* > 0.05, * *p* ≤ 0.05, ** *p* ≤ 0.01, *** *p* ≤ 0.001, **** *p* ≤ 0.0001).

To dissect the underlying mechanisms of the induced anti-leukemia effect and confirm the disruption of DOT1L recruitment to MLL target gene loci Hoxa9 and Meis1, we performed a chromatin immunoprecipitation coupled with qPCR (ChIP-qPCR) (Figure 2B). As expected, WT-DOT1L and RCR-DOT1L with an intact AF9-binding domain, were immunoprecipitated at the promoter region of both loci. However, in Δ10-DOT1L and I867A-DOT1L leukemia cells, we observed significantly decreased localization of DOT1L to these loci (Figure 2C). To validate that the loss of DOT1L recruitment was solely due to blocked recruitment by MLL-AF9, we also performed ChIP for Flag-MLL-AF9, showing its equal localization across the cell lines (Figure 2D). These results were also recapitulated at Intron 8 of Meis1 (Figure S4A). Next, we assessed the impact of disrupting the DOT1L and AF9 interactions on H3K79 methylation in MLL-AF9 leukemia cells expressing wild type or mutant DOT1L. Previously, we demonstrated that wild type DOT1L and Δ10-DOT1L with intact histone methyltransferase domains were able to restore H3K79me2 after the first round of plating in colony-forming unit assays, while RCR-DOT1L failed to restore H3K79me2 [38]. Similarly, we observed an overall slower change in global H3K79me2 in the PPIs mutant cells in comparison to cells expressing RCR-DOT1L. On day 2, Δ10-DOT1L and I867A-DOT1L showed modest changes in H3K79me2, while the DOT1L RCR mutation led to a global loss of H3K79me2. By day 3, all engineered cell lines showed no detectable H3K79me2 (Figure 3A,B). Thus, different kinetic rates of histone methylation changes were observed for global H3K79me2 levels when comparing constructs with impaired AF9-binding sites. Δ10-DOT1L and I867A-DOT1L showed a modest decrease in this epigenetic mark, whereas RCR-DOT1L cells show a complete loss of H3K79me2 on day 2. However, as expected, a significant decrease in H3K79me2 was identified on target genes, specifically at the Hoxa9 and Meis1 promoter regions, as well as intron 8 of Meis1 in all cell lines (Figure 3B and Figure S4B). Collectively, these findings provide evidence that the H3K79me2 marks on Hoxa9 and Meis1 are significantly decreased as a result of blocking DOT1L recruitment by disrupting its interactions with the MLL-AF9 onco-fusion protein, leading to inhibition of cellular proliferation.

We further characterized the cause of proliferative defects in these cells and evaluated well-established mechanisms of cell death when DOT1L’s function is genetically or pharmacologically inhibited [20,24,44]. Knowing that DOT1L primarily drives Hoxa9 and Meis1 expression for the initiation and maintenance of MLL-AF9 leukemia, we expected that with decreased H3K79me2, we would see a profound decrease in expression of MLL target genes [45,46,47,48]. Indeed, we observed a significant decrease in Meis1 and Hoxa9 expression in Δ10-DOT1L and I867A-DOT1L leukemia cells, comparable to the changes induced by complete loss of DOT1L (empty vector) or by DOT1L enzymatic inhibition (RCR-DOT1L). Importantly, these results correlated well with decreased H3K79me2 and blocked recruitment of DOT1L at both target loci (Figure 3C,D and Figure S5). Several studies showed that upon DOT1L enzymatic inhibition, cells harboring the MLL-AF9 fusion protein are driven to cell differentiation and apoptosis [24,44]. Thus, we explored if our constructs would recapitulate this phenotype in cell lines harboring the AF9-DOT1L PPI mutants. Wright-Giemsa staining revealed alterations in cell morphology from leukemic myeloblasts to differentiated cells when blocking DOT1L recruitment. This was further confirmed via concurrently increased expression of the monocyte differentiation marker CD14, and decreased c-Kit (CD117), a hematopoietic stem/progenitor marker, in Δ10-DOT1L and I867A-DOT1L cells in a similar manner as enzymatic inactivation of DOT1L (RCR-DOT1L) (Figure 3E). Altogether, these data provide functional confirmation of the importance and critical role of DOT1L recruitment in MLL-AF9-driven leukemogenesis, validating these protein-protein interactions as a potential therapeutic target in MLL-rearranged leukemia.

### 2.3. The Role of DOT1L, Its Enzymatic Activity and AF9-Binding Site in Non-Leukemic Hematopoiesis

**Figure 4:**
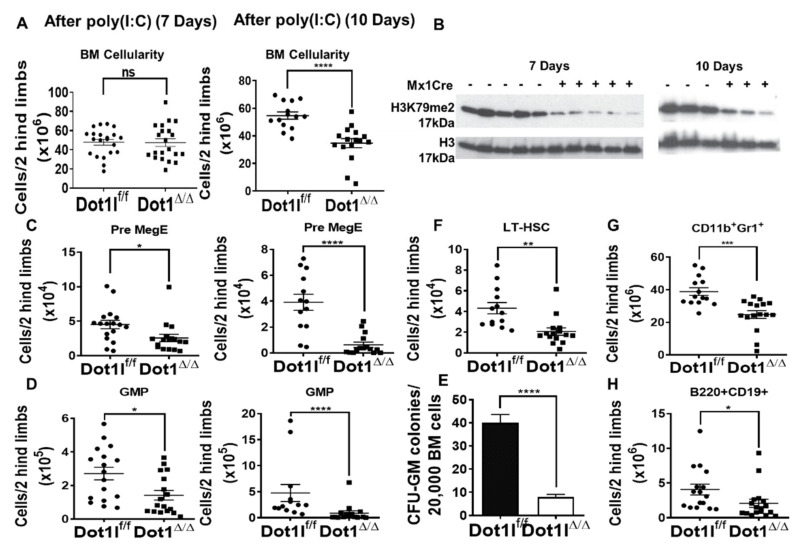
DOT1L inactivation in the adult bone marrow leads to rapid depletion of myeloid progenitors, followed by decrease in the numbers of hematopoietic stem cells (**A**) (left) Bone marrow cellularity in Dot1l^f/f^ Mx-Cre- and Dot1l^f/f^ Mx-Cre+ mice 7 days post poly(I:C) induction (n = 21 mice/group, pooled from 5 independent experiments; mean +/- SEM). (right) Bone marrow cellularity decreases in Dot1l^f/f^ Mx-Cre+ mice 10 days post poly(I:C) induction. (n = 14–16 mice/group, pooled from 3 independent experiments; mean +/- SEM). (**B**) Western blot shows decreased H3K79me2 in total BM at day 7 (left) and day 10 (right); (**C**) Flow cytometric analysis quantification shows that Pre MegE progenitors (CD150^+^CD105^-^CD16/32^-^CD41^-^LK) are significantly decreased as early as day 7 (left) and continue to be depleted by day 10 (right). (**D**) Quantification of flow cytometric analysis showing that granulocyte-macrophage progenitors (GMP) (CD16/32^+^CD150^-^CD41^-^LK) are significantly decreased as early as day 7 (left) and continue to decrease through day 10 (right). (**E**) Myeloid colony formation by wild-type vs Dot1l-null BM in CFU-GM assays (n = 8 mice/group with technical triplicates; mean +/- SEM; pooled from 2 experiments) showing a lack of colony formation potential in Dot1l ^Δ/Δ^ mice in comparison to Dot1l WT. (**F**–**H**) Profound decrease in LT-HSC, CD11b^+^Gr1^+^ BM myeloid cells, and B220^+^ CD19^+^B cells at day 10 post poly (I:C). (ns *p* > 0.05, * *p* ≤ 0.05, ** *p* ≤ 0.01, *** *p* ≤ 0.001, **** *p* ≤ 0.0001).

Studies of constitutive and conditional Dot1l knockout mice models show that Dot1l is essential for embryonic development, as well as prenatal and postnatal hematopoiesis [35,36,37]. Here, we assessed the impact of Dot1l loss on steady-state hematopoiesis in comparison to inhibition of DOT1L’s enzymatic activity and disruption of the protein-protein interactions between DOT1L and MLL-AF9. Understanding these effects is important to predict the potential side effects of profound inhibition of DOT1L enzymatic activity as well as blocking its recruitment by AF9 onco-fusion protein. In past studies, the effects of Dot1l inactivation became apparent as early as 2 weeks post excision in vivo, showing a rapid loss of all three lineage populations and decreased hematopoietic stem and progenitor populations [35,36,49]. However, persistent unexcised progenitors could have influenced the magnitude of the functional effects. Here, we aimed to identify the early events following highly efficient Dot1l loss in adult murine hematopoiesis. To achieve a high degree of gene inactivation and avoid compensation by undeleted progenitors, we introduced a type I interferon-inducible Mx1-Cre allele to the Dot1lf/f background. The Mx1-Cre transgene encodes a Cre recombinase that yields robust excision of floxed genes upon poly(I:C) injection in vivo, especially within the hematopoietic system [50,51]. Mice were administered poly(I:C) with 3 subsequent injections and sacrificed 7 days after the initial injection to inactivate Dot1l in the hematopoietic system. Bone marrow (BM) cellularity showed no significant differences between Dot1l-WT and Dot1l-deficient mice up to 1 week after Dot1l deletion. In contrast, on day 10 post-excision, we started to observe a significant decrease in total BM cellularity (Figure 4A). We observed complete Dot1l excision upon poly(I:C) injection in Mx1-Cre+ mice (Figure S6A), together with decreased H3K79 methylation at days 7 and 10 (Figure 4B), confirming that Dot1l was efficiently inactivated in these mice.

We next carefully examined the impact of Dot1l excision on hematopoietic stem cell and progenitor populations. For long-term hematopoietic stem cells (LT-HSCs) identified by the CD150+CD48– Lineage–Sca-1+c-Kit+ (LSK) phenotype and for the LSK cell population in general, there was no significant initial alteration in total cell numbers in the Dot1l-deficient mice at day 7 (Figure S6B) [52]. Downstream myeloid progenitor subsets were also evaluated by fractionating the Lineage-c-Kit+ (LK) compartment with additional markers (CD41, CD105, CD150, and CD16/32) [53]. We observed a significant decrease in the number of Pre-Megakaryocyte-Erythroid progenitors (Pre-MegE: CD41-CD16/32-CD150+ LK) and Granulocyte Macrophage Progenitors (GMP: CD41-CD16/32+ LK) in Dot1l-deficient mice (Figure 4C,D), as well as a significant decrease in CFU-GM colony-forming activity from Dot1l-deficient in comparison to Dot1l wild-type bone marrow (Figure 4E). These findings indicate that Dot1l loss affects hematopoietic progenitor function even ahead of its effects on HSC maintenance.

Since there was no initial alteration in HSCs, but a clear rapid depletion of progenitor cells as early as 7 days post Dot1l excision, we evaluated hematopoiesis at a later time point. In contrast with day 7 results, day 10 showed significant changes in stem cell and progenitor compartments. Dot1l-deficient mice had significant depletion in LK progenitors, LSK progenitors, LT-HSCs, and Pre-GM cells (Figure 4F and Figure S7A,B). Pro-Erythroid and Pre-CFU-E cells were also trending downward in numbers (Figure S7B). Furthermore, bone marrow CD11b+Gr1+ myeloid and B220+CD19+ B cells significantly decreased by day 10 (Figure 4G,H). These data are consistent with a profound effect of Dot1l loss on a broad range of primitive hematopoietic stem and progenitor cells.

We next evaluated whether inhibiting DOT1L recruitment by AF9 or blocking DOT1L enzymatic function have differential effects in the context of non-leukemic hematopoiesis. WT-DOT1L, Δ10-DOT1L, I867A-DOT1L, and RCR-DOT1L retroviral constructs were transduced ex vivo into Mx-Cre+ Dot1l f/f B6-CD45.2 mouse donor cells, in addition to a NUP98-HOXA10HD-IRES-mCherry construct. Introduction of the NUP98-HOXA10HD fusion protein was used to enhance the proliferation of donor HSCs ex vivo after retroviral transduction, based on past work showing that NUP98-HOXA10HD can dramatically increase HSC expansion without inducing transformation [54,55]. Indeed, cells transplanted in the absence of NUP98-HOXA10HD allowed for frequent host-derived reconstitution after *Dot1l* excision, which prevented efficient structure-function analysis of transduced donor-derived cells (not shown). First, we verified that NUP98-HOXA10HD could not rescue by itself the maintenance of bone marrow HSCs after *Dot1l* excision. We used a dual color readout system where cells derived from Mx1-Cre+ Dot1l f/f B6-CD45.2 and Mx1-Cre- Dot1l f/f B6-CD45.2 were transduced with either NUP98-HOXA10HD-IRES -mCherry or Nup98HoxA10-IRES-eGFP. These 4 cell populations were then mixed into two complementary groups with Cre+ and Cre- hematopoietic stem cells expressing complementary mCherry or eGFP-tagged NUP98-HOXA10HD constructs. These two groups were transplanted into WT B6-CD45.1 recipient mice and used for endpoint analysis after Dot1l excision (Figure S8A). Hematopoietic progenitors and myeloid cells from the Mx1-Cre+ Dot1lf/f B6-CD45.2 mice did not persist in the presence of the NUP98-HOXA10HD fusion protein regardless of the mCherry vs. eGFP reporter construct (Figure S8B,C). Thus, non-leukemic hematopoiesis supported by NUP98-HOXA10HD, remains highly sensitive to *Dot1l* loss and represents a good model to study the ability of DOT1L mutants to sustain hematopoiesis.

**Figure 5:**
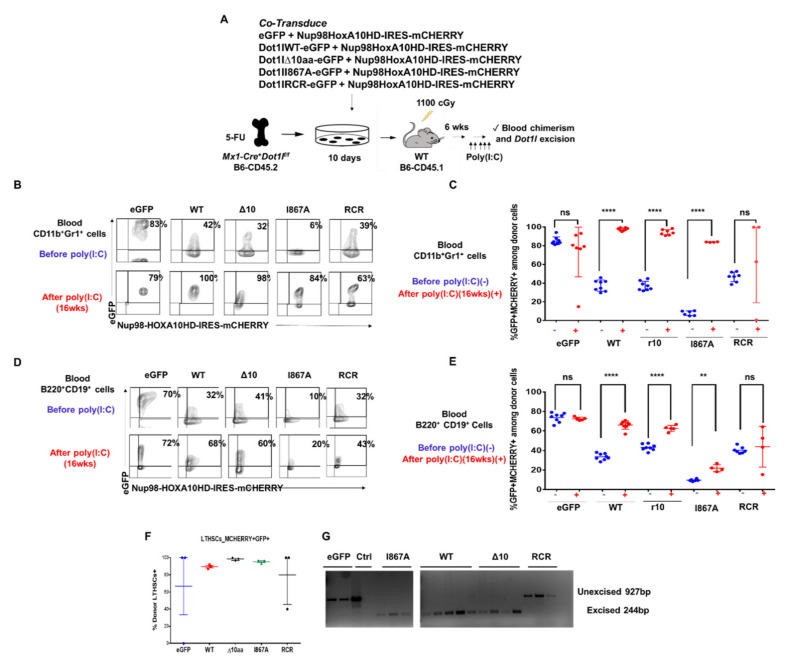
DOT1L’s enzymatic activity is essential in hematopoiesis, but its AF9-binding domain is dispensable. (**A**) Experimental approach. BM harvested from 5-FU-treated Mx-Cre^+^Dot1l^f/f^ B6-CD45.2 donors was co-transduced ex vivo with MSCV-based constructs expressing NUP98-HOXA10HD IRES-mCherry and eGFP alone vs. eGFP + DOT1L-WT, DOT1L-D10, DOT1L I867A or DOT1L-RCR. After ex vivo expansion, these cells were transplanted into irradiated B6-CD45.1 recipients. After hematopoietic reconstitution, poly(I:C) was administered to inactivate endogenous *Dot1l*, followed by analysis of chimerism and Dot1l excision; (**B**) Representative examples of flow cytometric data. Numbers show the percentage of mCherry^+^eGFP^+^ cells among blood myeloid cells; (**C**) Flow cytometric analysis of blood CD45.2^+^ donor-derived CD11b^+^Gr1^+^ myeloid cells for mCherry and eGFP expression, before (blue) and 16 wks after (red) poly(I:C) (5 i.p. doses). Data from individual mice are shown. Increased representation of cells expressing DOT1L-WT, DOT1L-D10, and DOT1L-I867A, but not eGFP or DOT1L-RCR was seen after poly(I:C); (**D**) Flow cytometric analysis of blood CD45.2^+^ donor-derived B220^+^CD19^+^ myeloid cells for mCherry and eGFP expression, before (blue) and 16 wks after (red) poly(I:C) (5 i.p. doses). Data from individual mice are shown. Increased representation of cells expressing DOT1L-WT, DOT1L-D10, and DOT1L-I867A, but not eGFP or DOT1L-RCR was seen after poly(I:C); (**E**) LT-HSCs after poly(I:C) administration at the termination of the study. DOT1L-WT, DOT1L-D10 and DOT1L-I867A recipients have LT-HSCs that are comprised mostly of eGFP+ donor cells; (**F**) PCR analysis of *Dot1l* excision in sort-purified blood CD45.2^+^mCherry^+^eGFP^+^ myeloid cells 20 weeks after poly(I:C) administration. (**G**) DOT1L-WT, DOT1L-D10 and DOT1L-I867A recipients had ongoing myelopoiesis with fully excised *Dot1l*, while eGFP and DOT1L-RCR recipients had selected cells escaping *Dot1l* inactivation. (ns *p* > 0.05, ** *p* ≤ 0.01, **** *p* ≤ 0.0001).

After ex vivo expansion, CD45.2+ hematopoietic progenitors carrying NUP98-HOXA10HD plus one of the DOT1L constructs were transplanted into irradiated B6-CD45.1 recipient mice allowing for a clear delineation of host and recipient-derived cells. After hematopoietic reconstitution, poly(I:C) was administered (Figure 5A). Blood from the recipient mice was collected 16-weeks post poly(I:C) injection and analyzed by flow cytometry for CD45.2+ donor-derived CD11b+Gr1+ myeloid cells with both mCherry and eGFP colors (Figure 5B). Rates of transduction by the DOT1L constructs differed between the groups, as shown by the various percentages of eGFP+mCherry+ at baseline before poly(I:C) injection. However, the presence of WT-DOT1L, Δ10-DOT1L, and I867A-DOT1L in donor cell populations led to an increased representation for eGFP+mCherry+ cells in the blood after the loss of endogenous Dot1l, indicating that these cells were selected based on the exogenous DOT1L expression, and thus that these mutant forms of DOT1L could rescue the function of endogenous Dot1l. In contrast, empty vector and RCR-DOT1L did not induce a systematic increase in eGFP+ cells from 2 to 16 weeks post poly(I:C) injection (Figure 5C and Figure S9A). This trend was also observed in circulating B cells (Figure 5D,E and Figure S9B). At the termination of the experiment, we observed that the LT-HSC cell population was made up entirely of donor–derived WT-DOT1L, Δ10-DOT1L, and I867A-DOT1L, but not RCR-DOT1L and eGFP cells (Figure 5F). We then tested these mice for persistent excision of endogenous Dot1l to account for the selection of rare escapees. Cells that retained endogenous Dot1l were able to sustain hematopoiesis in eGFP and RCR-DOT1L mice. Indeed, these two groups of mice expressing empty vector and RCR-DOT1L cells had not maintained excision of endogenous Dot1l as observed by PCR analysis at the termination of the experiment (Figure 5G). In contrast, short-lived myeloid cells harvested at the end of the experiment still had an excised endogenous Dot1l locus when rescued with WT-DOT1L, Δ10-DOT1L, or I867A-DOT1L. Thus, we observed a profound impact on leukemogenesis when DOT1L recruitment to AF9 was targeted, with no effects on the maintenance of non-leukemic hematopoiesis in adult mice. We acknowledge that our observations have been made in NUP98-HOXA10HD-transduced hematopoietic cells for technical reasons, rather than in normal hematopoietic cells. However, NUP98-HOXA10HD did not bypass the requirement for *Dot1l* in hematopoiesis. Altogether, these findings validate the inhibition of DOT1L-AF9 protein-protein interactions as a therapeutic strategy in leukemic cells that overcomes the on-target side effects of blocking DOT1L enzymatic activity in hematopoiesis.

## 3. Discussion

Preclinical studies using genetic or small molecule inhibitors demonstrated that DOT1L methyltransferase activity is required for MLL-fusion–mediated leukemogenesis and validated DOT1L as a promising therapeutic target for the treatment of *MLL*-rearranged leukemia [15,16,19,20,56,57]. EPZ-5676, pinometostat, the first-in-class inhibitor of DOT1L histone methyltransferase activity, showed partial responses in a Phase 1 clinical trial with pharmacodynamic evidence of DOT1L inhibition in leukemic blasts [26,27]. Based on these results, further clinical investigation of combination treatments has been considered [58].

EPZ-5676, and other reported DOT1L inhibitors bind to the catalytic domain and affect DOT1L globally [24,59,60,61]. Since DOT1L is the only known H3K79 histone methyltransferase and is involved in fundamental biological processes, we need to be cautious about potential side effects. Thus, there is an unmet need to identify and validate new strategies for selectively inhibiting DOT1L activity in MLL-complexes, while preserving its function in other tissues.

About half of the AML patients with MLL-driven leukemia are diagnosed with only 4 different *MLL* translocations: *MLL-AF4*, *MLL-AF9*, *MLL-ENL*, and *MLL-AF10*. Emerging findings from a number of groups suggest that partners of the common MLL fusions, including MLL-AF4, MLL-AF9, and MLL-ENL, are part of multiprotein complexes involved in transcriptional activation/elongation [62,63]. One of the mechanisms by which MLL fusion proteins activate target genes is via the recruitment of methyltransferases during transcription to maintain an open chromatin conformation. It is known that the recruitment of DOT1L results in hypermethylation of H3K79 on the prominent MLL fusion downstream target loci *HoxA9* and *Meis1* [64]. Genome-wide analysis revealed a distinct pattern of H3K79 methylation in human MLL-rearranged primary leukemia samples compared with normal pro-B cells and leukemia with other abnormalities [18,65,66]. Pre-clinical in vitro and in vivo studies have demonstrated a strong functional interconnection between complexes formed by MLL fusion proteins and DOT1L [34,64]. These findings illustrate the central role of DOT1L recruitment and H3K79 methylation in leukemogenesis by controlling transcription of hematopoietic genes and implicate PPIs between DOT1L and MLL oncogenic fusion proteins as a potential therapeutic target. We and others have characterized the PPIs between MLL-AF9/ENL fusion proteins and DOT1L on biochemical, structural and functional levels [9,38,39].

In this study, we further explore the therapeutic value of targeted disruption of the DOT1L recruitment by MLL-AF9 onco-fusion protein in AML and elucidate the role of this protein-protein interaction in non-leukemic hematopoiesis. For this purpose, we established, characterized, and used MLL-AF9 transformed leukemia cell lines transduced with several different DOT1L constructs, including wild type and mutants, to disrupt either PPIs or enzymatic activity. Consistent with the essential role of DOT1L and H3K79 methylation as a critical mechanism to initiate and maintain leukemogenesis in MLL-rearranged leukemia [15,16,17], the MLL-AF9 interaction with DOT1L plays a crucial role in MLL-AF9 transformed leukemia cells, in a similar manner to that seen with inhibition of DOT1L enzymatic activity (Figure 2). Using established stable conditional knockout *Dot1l* murine lines that express both HA-tagged wild type or mutant DOT1L proteins, and Flag-tagged MLL-AF9, our work demonstrates that by blocking the PPIs between MLL-AF9 and DOT1L by a single point mutation (1867A), we can phenocopy enzymatic inhibition of DOT1L that leads to impaired leukemic cell growth, and differentiation, (Figure 3). As expected, DOT1L mutant proteins, Δ10-DOT1L and I867A-DOT1L, disrupted the MLL-AF9 and DOT1L interaction, thus blocking DOT1L recruitment to *Hoxa9* and *Meis1* target gene loci. This leads to a significant decrease in H3K79me2 in the promoter regions of these genes. Importantly, evaluation of the global H3K79 methylation provided insights into the slower dynamic rate of the H3K79me2 loss in these cells, in comparison to RCR-DOT1L, which inhibits enzymatic activity but maintains an intact AF9 binding site. These results suggest that disruption of the interaction between DOT1L and AF9/ENL might provide selective targeting of MLL fusion proteins and bring advantages and fewer adverse effects in comparison with the enzymatic inhibition of DOT1L, given the genome-wide role of DOT1L in the regulation of transcription and its effect on many genes. Overall, our data point to a potential novel therapeutic strategy for the treatment of patients bearing MLL translocations by blocking the PPIs and recruitment of DOT1L by MLL-AF9.

To identify the potential therapeutic benefits of this therapeutic strategy, we investigated the effect of DOT1L mutants with an impaired AF9 binding site on non-leukemic hematopoiesis. Several reports described adverse effects of DOT1L loss on normal hematopoiesis in mice after two or more weeks of *Dot1l* excision [35,36]. Our studies, focused on assessing the early effects on normal hematopoiesis and identifying the cell populations that are the most affected by DOT1L loss in normal bone marrow. We found that as early as one week after DOT1L loss, myeloid progenitor cells and other progenitor populations were most sensitive to *Dot1l* loss, while total bone marrow cellularity remained normal. By day 10, mice started to lose total bone marrow cellularity in comparison to mice with preserved DOT1L. There was also a significant loss of LT-HSCs cells as well as progenitor cell populations. These findings are in agreement with previous studies showing profound effects on progenitor cell populations in mice lacking DOT1L at later time points [35,36]. The rapid and striking effects on adult hematopoiesis upon *Dot1l* inactivation point towards the potential of on target side effects on hematopoiesis when enzymatically inhibiting DOT1L. Interestingly, in our studies using Δ10 and I867A DOT1L mutants, which have an impaired AF9 binding site that disrupts DOT1L recruitment, we demonstrated a potent reconstitution of the non-leukemic bone marrow in these mice. On the contrary, the RCR and control empty vector derived cells revealed impaired bone marrow reconstitution with strong selection for progenitors that escaped excision of endogenous *Dot1l*. We acknowledge the limitation that our studies were performed in NUP98-HOXA10HD-driven hematopoiesis because of technical considerations. Although use of NUP98-HOXA10HD did not bypass the requirement for *Dot1l* to sustain hematopoiesis, allowing us to establish a reliable in vivo structure function assay, our observations will need to be validated in normal hematopoiesis. Altogether, our data suggest that non-leukemic hematopoiesis can be sustained when the PPIs between MLL-AF9 and DOT1L are disrupted, demonstrating that significant therapeutic advantages might be afforded by selective targeting of these PPIs. In addition, these results conclusively provide evidence that DOT1L recruitment can be completely abolished by a single point mutant I867A. This also demonstrates a crucial role for this residue in hydrophobic protein-protein interactions and provides important insight for further drug discovery.

## 4. Materials and Methods

### 4.1. Fluorescence Polarization

Competitive FP binding experiments were performed in 96-well black, round-bottom plates (3792, Corning, Tewksbury, MA, USA) with serial dilutions of peptide or compounds, with a fixed concentration of MBP-AF9 (200 nM) and N-Flu-DOT1L (10 nM) in assay buffer (100 mM NaH_2_PO_4_, 150 mM NaCl, 0.1% β-ME, 0.01% Triton X-100) to a final volume of 125 μL. The polarization units were measured after mixing and incubating at room temperature for 3 h. Negative controls with MBP-AF9 and N-Flu-DOT1L probe only (0% inhibition) and positive controls with N-Flu-DOT1L probe only (100% inhibition) were included in the assay plate. IC50 values were determined by nonlinear regression fitting of the competitive curves using GraphPad Prism 5.0 (GraphPad Software, San Diego, CA, USA).

### 4.2. Mice

All experiments in mice were approved by the University of Michigan’s Committee for the Use and Care of Animals. C57BL/6.Ptprca (B6-SJL, CD45.1+) were bred in-house or obtained from Charles River (Frederick, MD, USA). C57BL/6 *Dot1lf/f* mice were obtained from Dr. Jay Hess [35] and crossed to *Mx1-Cre+* mice [51] for in vivo experiments, or to *Cre-ERT2* mice for in vitro experiments. Specifically, for *C57BL/6 Dot1lf/f Mx1 Cre+* mice, breeding was complicated by genetically linked loci on mouse chromosome 10, although *Dot1lf/+ Mx1-Cre+* progeny was eventually obtained and then intercrossed [67]. Dot1l excision was achieved using poly(I:C) (Cytiva, formerly Amersham//GE Healthcare, Marlborough, MA, USA; 50 μg i.p. every 2 days for 3–5 doses) in vivo or 4-hydroxytamoxifen (4-OHT) in vitro (Sigma-Aldrich; St Louis, MO, USA; 7.5 nM at experimental day 0 and day 2).

Genotypes of individual mice were confirmed by polymerase chain reaction (PCR). All experiments in mice were approved by the University of Michigan’s Committee for the Use and Care of Animals.

### 4.3. Cell Line Generation

Bone marrow from *Dot1lf/f CreER(T2)^+^* mice was harvested 4 days after i.p. injection of 150 mg/kg 5-fluorouracil (F6627, Sigma-Aldrich) and lineage-depleted using the EasySep Mouse Hematopoietic progenitor cell isolation kit (19856, StemCell Technologies, Vancouver, BC, Canada). Retroviruses were produced by transfecting *MSCV-neo-Flag-MLL-AF9* construct into PlatE cell line with Fugene 6 (E2691, Roche Diagnostics, Indianapolis, IN, USA). Fresh viral supernatant was used for transduction of the lineage depleted *Dot1lf/f CrER(T2)^+^* stem cells using two subsequent rounds of spinoculation. For spinoculation, cells were spun at ~2000 rcf in the presence of viral supernatant for 90 min at room temperature. Cells underwent neomycin selection for 6 days and were weaned off stem cell factor (SCF) 9 days after starting selection. These selected cells were then transduced 14 days following initial antibiotic selection using the same spinoculation method described above with one of the following vectors: *MigR*1 (empty vector), *MigR1-HA-DOT1L* (WT-DOT1L), *MigR1-HA-DOT1L* deletion aa865–874 *(Δ10-DOT1L)*, *MigR1-HA-DOT1L* I867A (I867A-DOT1L), and *MigR1-HA-DOT1L RCR* (RCR-DOT1L). Cells were left to recover for 3 days, then sorted for GFP positivity using a MoFlo Astrios FACS sorter (Beckman Coulter, Indianapolis, IN, USA). Cells were expanded and resorted to get a majority of GFP+ cells expressing the DOT1L constructs.

### 4.4. Cell Growth

Cell numbers were assessed using a hemocytometer. Cells were treated with either 7.5 nM 4-OHT or an equivalent concentration of solvent (100% ethanol) on day 0 and day 2 to achieve excision of *Dot1l*, followed by daily counting.

### 4.5. Chromatin Immunoprecipitation

ChIP experiments were performed by treating the *Dot1lf/f CreER-(T2)* MLL-AF9 cell lines carrying the reintroduced DOT1L WT/mutant constructs or empty vector control with 7.5 nM 4-OHT for 3 days. Briefly, 3 × 10^7^ cells were crosslinked with 1% formaldehyde, lysed with 1% SDS lysis buffer (50 mM Tris HCl pH 8, 10 mM EDTA, 1 Roche mini tablet of protease inhibitors diluted in 1 mL (for 10 mL), 1% SDS) and sonicated on a Biorupter Pico sonication device (Diagenode, Denville, NJ, USA). Lysates were diluted in ChIP dilution buffer (1% Triton, 1.2 mM EDTA, 167 mM NaCl, 16.7 mM Tris HCl pH8) and precleared with appropriate mouse or rabbit IgG beads. Cleared lysates were immunoprecipitated with anti-HA (ab1424, Abcam, Cambridge, UK), anti-Flag (ThermoFisher, Waltham, MA, USA), anti-H3K79me2 (ab3594, Abcam), anti-H3 (24834, Abcam) using protein A/G agarose beads (sc-2003, SantaCruz, Biotechnology, Dallas, TX, USA). The IPs were washed with a low salt (150 mM NaCl, 0.1% SDS, 20 mM Tris–HCl pH8, 2 mM EDTA, 1% Triton X-100), a high salt buffer (500 mM NaCl, 0.1% SDS, 1% Triton X-100, 20 mM Tris–HCl pH8, 2 mM EDTA) and a stringent lithium chloride buffer (0.25 M LiCL, 1% IGEPAL CA-630, 10 Mm Tris–HCl pH8, 1 mM EDTA, 1% sodium deoxycholate). Protein-DNA complexes were eluted in 1% SDS, crosslinked in high salt and treated with RNaseA and Proteinase K. DNA was purified with a PCR purification kit (Qiagen, Germantown, MD, USA). qPCR was performed using the SYBR-green mastermix. Primer sets used were: *Meis1*_promoter_F-5′TCAAAGTGACAAAATGCAAGCA3′; *Meis1*_promoter_R-5′-CCCCCCGCTGTCAGA AG-3′; *Hoxa9*_promoter_F-5′-TGACCCCTCAGCAAGACAAAC-3′; *Hoxa9*_promoter_R- 5′-TCCCGCTCCCCAGACTG-3′.

### 4.6. Co-Immunoprecipitation

Cells expressing both HA-DOT1L constructs and Flag-MLL-AF9 were collected and lysed using BC-300 lysis buffer: 20 mM Tris-HCl pH 8.0, 300 mM KCl, 1 mM EDTA, 10% glycerol, 0.1% NP-40 and 1x protease inhibitor cocktail (BioMake, Houston, TX, USA). The lysate was pre-cleared for 2 h in Mouse IgG Agarose (Sigma-Aldrich). The cell lysates were immunoprecipitated with anti-Flag M2 magnetic beads (M8823, Sigma-Aldrich) at 4 °C for 2 h. After incubation, the beads were washed extensively, boiled in SDS loading buffer and analyzed by western blotting using anti-Flag M2 (Sigma-Aldrich) and anti-HA tag (ab1424, Abcam) antibody.

### 4.7. Histone Methylation

Cells were harvested and processed using a previously reported method [68]. Histones were quantified using a Bradford assay and normalized for SDS page electrophoresis. Anti-H3K79me2 (ab3594, Abcam) and anti-H3 (ab1791, Abcam) loading controls were used for probing.

### 4.8. Gene Expression

Cells were harvested from the cell proliferation assay on day 3. RNA was isolated, and cDNA was synthesized using Superscript III reverse transcriptase kit (18080085, Invitrogen, Carlsbad, CA, USA). Quantitative real-time PCR was performed using SyberGreen master mix (4344463, Applied Biosystems, Foster City, CA, USA) and primers for Hoxa9, Meis1, GAPDH, and β-actin. Data was analyzed using the 2-∆∆Ct method. Expression was normalized to *GAPDH* expression and was performed in triplicate.

### 4.9. Differentiation

Cells were harvested from the cell proliferation assay on day 3 and cytospun onto microscope slides. Cells were then stained with Wright-Giemsa stain (EMD Millipore, Burlinigton, MA, USA) and mounted on a slide with a coverslip using Permount (Fisher Scientific Waltham, MA, USA) mounting medium. Images were captured using bright field condition with an IX83 inverted microscope (Olympus, Center Valley, PA, USA). Cells were stained with anti-c-Kit, anti-CD14 and isotype control antibodies (BD Pharmingen, San Jose, CA, USA and Biolegend, San Diego, CA, USA) and processed in a LSRII Flow Cytometer (BD Biosciences, San Jose, CA, USA). Data was analyzed using FlowJo X (Tree Star, San Carlos, CA, USA).

### 4.10. Flow Cytometry

Single cell suspensions were prepared from bone marrow or blood, followed by red blood cell lysis (ACK buffer, Cambrex, Walkersville, MD, USA). The following antibodies were from BioLegend: anti-CD3 (100304, 100308), CD4 (116006), CD8α (100708), CD11b (101208, 101204), CD11c (117308, 117304), CD16/32 (101317), CD19 (115508, 115520), CD48 (103404, 103412), CD105 (120411), CD150(SLAM) (115904, 115914), Ly-6G/Ly-6C(Gr-1) (108408, 108404, 108445), CD45R/B220 (103208, 103224, 103204), NK-1.1 (108708), TCRβ (109208, 109212, 109204), TCRγ/δ (118103, 118108), CD117(c-kit) (105826), Ly-6A/E(Sca-1) (108128), CD45.1 (110724), CD45.2(109836). The following antibodies were from eBiosciences: CD8α (13-0081), CD19 (13-0193), CD41a (17-0411), Ly-6A/E(Sca-1) (45-5981), NK1.1 (13-5941). The following reagent was from BD Biosciences: PE-CF594 Streptavidin (562284). We used the following antibody cocktail to exclude Lineage-positive cells: anti- CD3 (100304, 100308, BioLegend), CD8α (100708, BioLegend; 13-0081; eBiosciences), CD11b (101208, 101204, BioLegend), CD11c (117308, 117304, BioLegend), CD45R/B220 (103208, 103204, BioLegend), CD19 (115508, BioLegend; 13-0193, eBiosciences), TCRβ (109208, 109204, BioLegend), TCRγ/δ (118103, 118108, BioLegend), Ly-6G/Ly-6C(Gr-1) (BioLegend 108408, 108404), NK-1.1 (BioLegend 108708, eBiosciences 13-5941), and Ter119/Erythroid Cells (116208, 116204, BioLegend). Ter119/Erythroid Cells was omitted when analyzing erythroid progenitor populations. Ki67 staining was achieved using the BD Pharmingen PE Mouse Anti-Ki-67 Set (556027, BD Biosciences). Analysis was performed on a BD LSRFortessa flow cytometer (BD Biosciences) and sorting on FACSAria II/III flow cytometer (BD Biosciences). Dead cells were excluded with 4′6-diamidino-2-phenylindole (D9542, Sigma Aldrich) or with Zombie Aqua Fixable Viability Kit (423102, BioLegend). Files were analyzed in FlowJo.

### 4.11. Retroviral Transduction and Bone Marrow Transplantation

Donor *Dot1lf/f Mx1-Cre+* CD45.2+ *and Dot1lf/f Mx1-Cre*- CD45.2+ mice were injected i.p. with 150 mg/kg 5-FU to enrich for cycling HSCs. Bone marrow was harvested 4 days after 5-FU treatment and stimulated with SCF, IL-3 and IL-6 overnight, as described [41]. To achieve potent hematopoietic engraftment without transformation, *Mx-Cre^+^Dot1l f/f* B6-CD45.2 and *Dot1lf/f Mx1-Cre-* CD45.2 cells were expanded ex vivo for 10 days after co-transduction with MSCV-based constructs expressing *eGFP* plus *DOT1L-WT*, *DOT1L-*∆*10*, *DOT1L-I867A* or *DOT1L-RCR* and NUP98-HOXA10HD-IRES-mCherry so that we could color trace the presence of NUP98-HOXA10HD in cells via mCherry, in the presence or absence or exogenous DOT1L constructs. In selected control experiments, both NUP98-HOXA10HD-IRES-mCherry and NUP98-HOXA10HD-IRES-eGFP constructs were used. On the day of transplant, 6–8-week-old B6-CD45.1 mice were lethally irradiated (5.5 Gy twice 4–6 h apart, Cesium-137 source). Three hours after the second irradiation dose, mice were transplanted with expanded and retrovirally transduced progenitors. Mice were left to rest for 6 weeks before poly(I:C) injections started. Blood was obtained through retroorbital bleeding and transferred to EDTA-treated tubes. Complete blood counts were determined using the Advia 120 Hematology System (Siemens, Malvern, PA, USA). Flow cytometry was performed as described above, combining eGFP and mCherry fluorescence with staining for selected surface markers.

### 4.12. Western Blot

Whole bone marrow was lysed with 2x Laemmli Sample Buffer (BioRad, Hercules, CA, USA) plus 2-mercaptoethanol (Sigma, St. Louis, MO, USA). For assessment of H3K79 di-methylation, lysates from 10–15 × 10^5^ bone marrow cells were probed with anti-H3K79me2 (Abcam, ab3594, 1:1000 dilution) and anti-H3 (ab1791, Abcam, 1:5000 dilution).

### 4.13. CFU-GM

20,000 bone marrow cells were plated per mL of Methocult GF M3534 in triplicate for each biological sample (Stemcell Technologies, Vancouver, BC, Canada). Colonies were counted 12 days later.

### 4.14. Statistical Analysis

Comparison of two means was performed with 2-tailed unpaired Student’s t-test. Welch’s correction was utilized when data did not fit a normal distribution.

## 5. Conclusions

**Figure 6:**
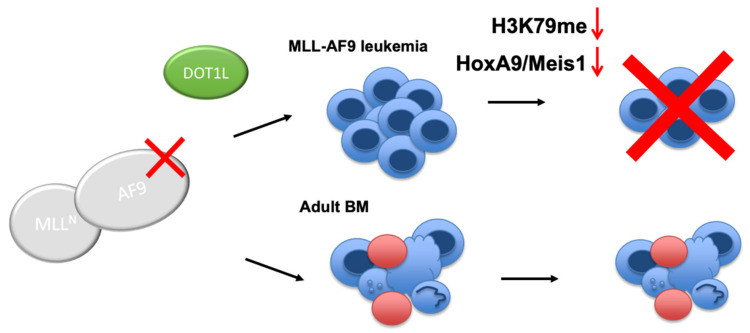
Proposed model for targeting the AF9-DOT1L interaction. Disrupting the AF9-DOT1L PPI downregulates H3K79me and *HoxA9* and *Meis1* expression leading to cell differentiation and cell death. In contrast, in non-leukemic bone marrow (BM), the AF9-DOT1L interaction is dispensable wherein hematopoietic stem and progenitor cells are sustained.

We have successfully developed the first class of peptidomimetics that target PPIs between DOT1L and MLL onco-fusion proteins, AF9 and ENL, based on the 10mer DOT1L peptide [40]. The findings of this study provide further evidence validating the rationale for a novel promising strategy that disrupts interactions between DOT1L and AF9 in MLL fusion protein-associated leukemia. We further propose that targeting these PPIs will offer therapeutic advantages over enzymatic inhibition of DOT1L in MLL-rearranged leukemia, which globally effects gene expression of which the long-term effects are not yet clear. By targeting the MLL-AF9-DOT1L interaction, DOT1L recruitment is blocked, leading to inhibition of the growth of MLL-AF9-driven leukemia cells, selective downregulation of MLL-target gene expression by decreasing H3K79 methylation, followed by differentiation. Importantly, the DOT1L enzyme activity remains intact to carry out its normal functions in the hematopoietic compartment (Figure 6). Our study provides a proof-of-concept for further development of nonpeptidic, cell-permeable compounds to inhibit DOT1L by targeting its recruitment and interactions with the MLL-fusion protein partners, AF9 and ENL, validating their therapeutic value in pre-clinical in vivo mouse models.

## Data Availability

Not applicable.

## Supplementary Materials

The following are available online at https://www.mdpi.com/2072-6694/13/4/642/s1, Figure S1: Validation of the expression and interaction of DOT1L constructs and MLL-AF9 in established murine cell lines, Figure S2: *Dot1L* excision is maintained throughout the course of the experiment, Figure S3: Assessing 7.5nM 4-OHT is toxicity and Cre induction in the DOT1L constructs and MLL-AF9 in established murine cell lines., Figure S4: ChIP at Intron 8 of *Meis1*, Figure S5: Raw qPCR data for *HoxA9/Meis1* gene expression. Figure S6: Day 7 of *Dot1l* inactivation shows early impacts some progenitor cell populations., Figure S7: HSCs and progenitors are depleted 10 days after *Dot1l* inactivation., Figure S8: Nup98HoxA10HD does not rescue DOT1L loss., Figure S9: The AF9-binding domain of DOT1L is dispensable for adult hematopoiesis.
