# Supplementary material for: Elucidating the Importance of DOT1L Recruitment in MLL-AF9 Leukemia and Hematopoiesis
Source: Cancers (Basel). 2021 Feb 5;13(4):642. doi: 10.3390/cancers13040642 (PMC7914713; doi:10.3390/cancers13040642)
Supplement: Supplementary file 1 [file cancers-13-00642-s001.pdf]

cancers

MDPI

Supplementary material

Elucidating the importance of DOT1L recruitment in MLL-AF9 leukemia and hematopoiesis

Sierrah M. Grigsby, Ann Friedman, Jennifer Chase, Bridget Waas, James Ropa, Justin Serio, Chenxi Shen, Andrew Muntean, Ivan Maillard and Zaneta Nikolovska-Coleska

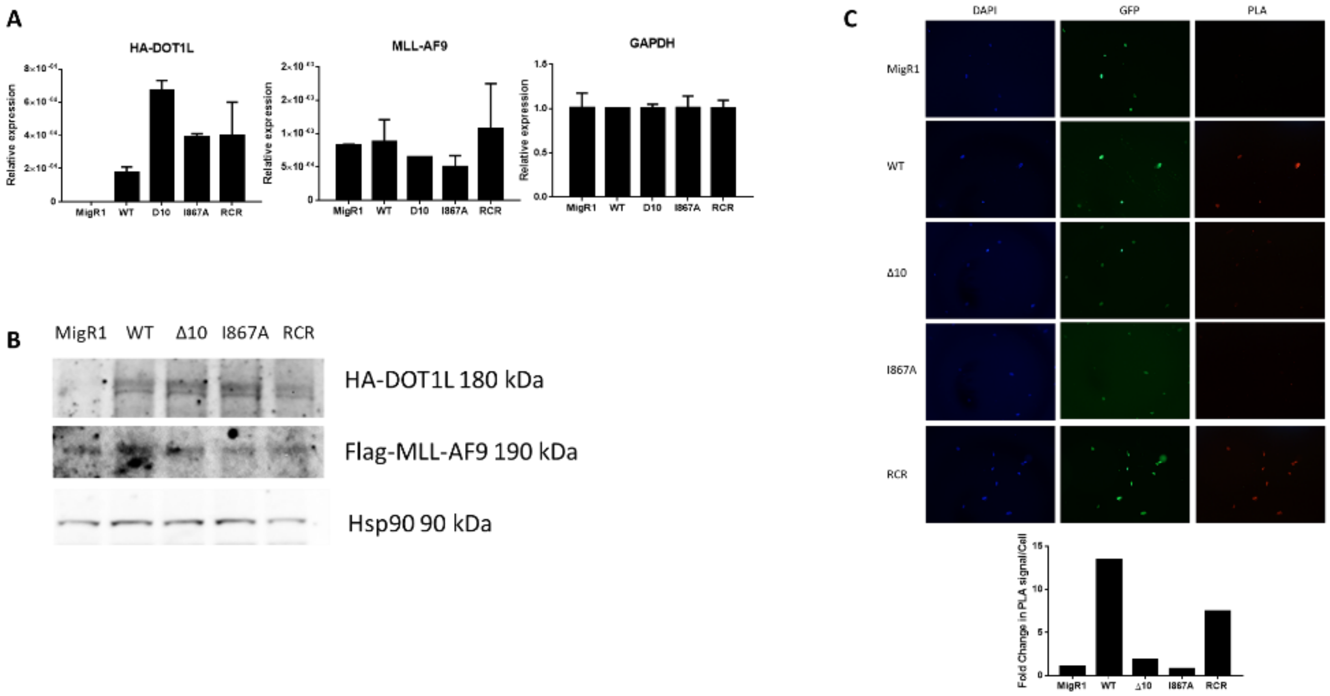

Figure S1. Validation of the expression and interaction of DOT1L constructs and MLL-AF9 in established murine cell lines. (A) Expression levels of HA-DOT1L constructs and MLL-AF9 in the established cell lines. Cells containing mutant constructs have a higher level of transcript present than WT; however, the expression of wild type is sufficient for rescuing the phenotype of endogenous DOT1L deletion. However, no significant difference in MLL-AF9 expression and corresponding GAPDH control. **(B)** Western Blot of lysate from MLL-AF9 cell lines with different DOT1L constructs showing that the HA-DOT1L and Flag-MLL-AF9 constructs are expressed and recognized by the HA and Flag antibodies that were using in subsequent experiments in the MLL-AF9 cell lines. **(C)** Proximity Ligation Assay (PLA) of the Flag tagged MLL-AF9 murine cell lines with MigR1-HA-DOT1L constructs and average quantification of PLA signal per cell. The cells were fixed with EtOH and incubated with HA and Flag antibodies to capture the AF9-DOT1L interaction. The first column shows the DAPI staining showing the presence of cells followed by the GFP that is showing the cells that is expressing the DOT1L constructs and the last column show the PLA signal that is observed when the two antibodies are in close proximity indicating a protein-protein interaction. (Olympus IX83 Inverted Microscope; Original magnification x200).

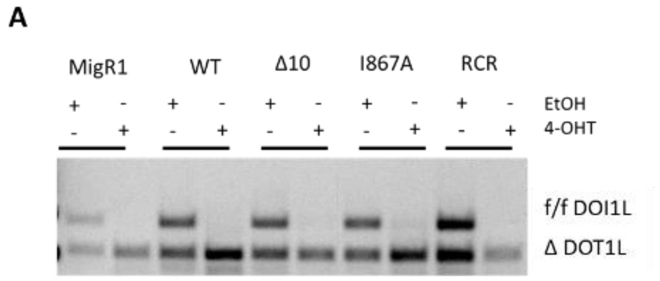

Figure S2. *Dot1L* excision is maintained throughout the course of the experiment (A) Genotyping of f/f DOT1L CreERT2 MLL-AF9 cell lines with DOT1L constructs showed excision of endogenous DOT1L for the duration of the experiment. The figure shows a representative genotyping at Day 3. Genotyping primers GAAGTTCCTATTCCGAAGTT and GAAC-CACAGGATGCTTCAG: f/f Dot1l allele (927bp) and ΔDOT1L allele (244bp)[1].

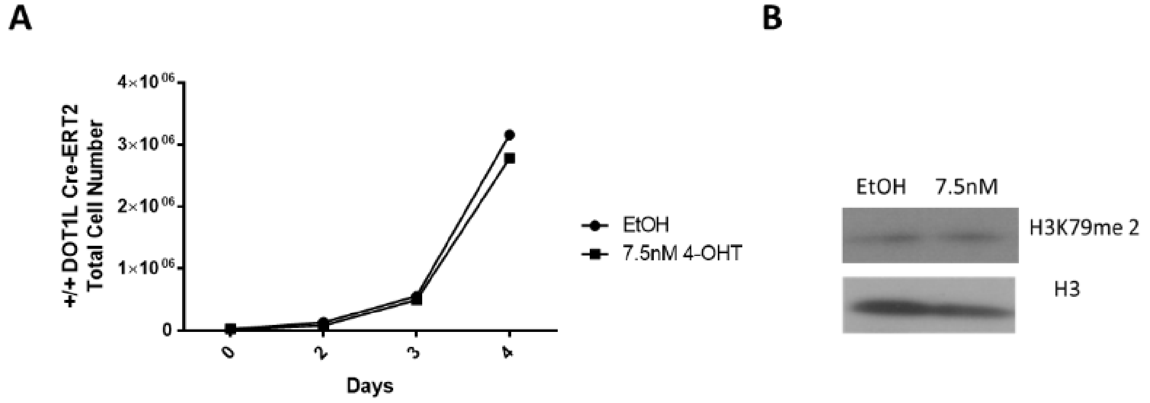

Figure S3. Assessing the toxicity of 7.5 nM 4-OHT and Cre induction in the DOT1L constructs and MLL-AF9 in established murine cell lines. (A) WT DOT1L CreERT2 MLL-AF9 cells were treated with 7.5 nM tamoxifen (4-OHT) showing no effect on cell proliferation (B) Western blot showing no effect on H3K79me2 after 4 days of 4-OHT treatment.

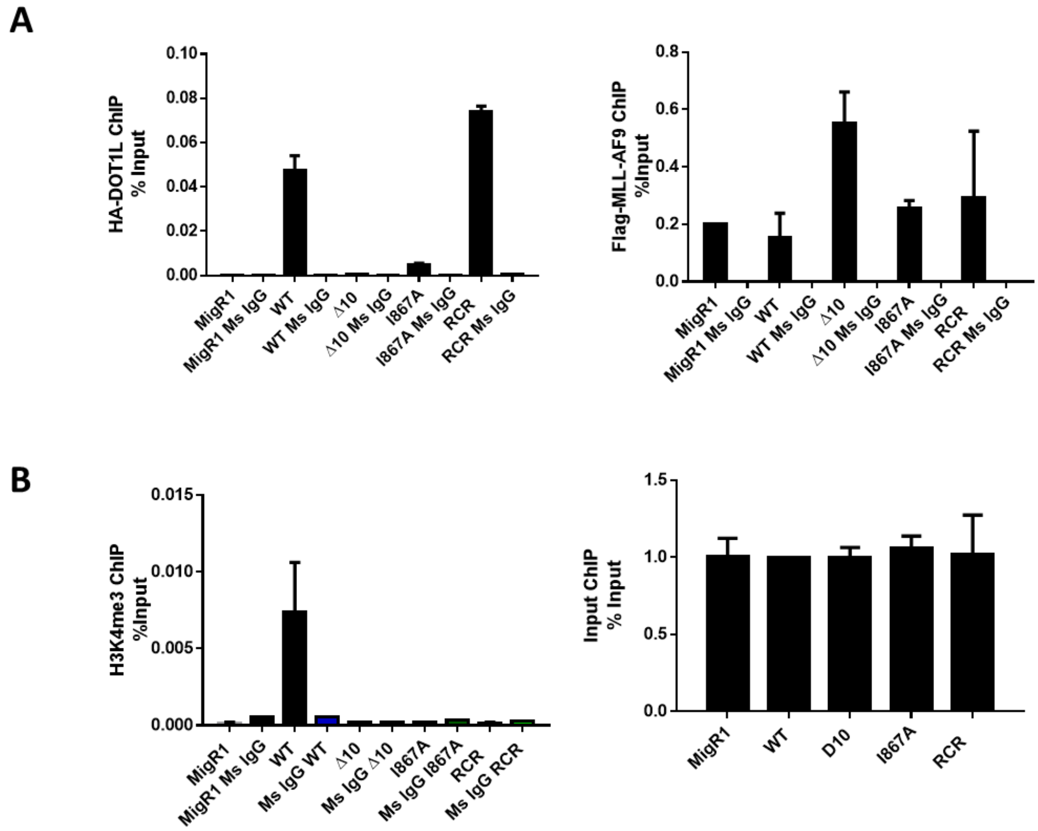

Figure S4. ChIP at Intron 8 of *Meis1*. (A) ChIP-qPCR using an HA antibody, DOT1L constructs were pulled down showing discretion of the MLL-AF9 and DOT1L interaction at Intron 8 of *Meis1*. Only WT and RCR constructs with the intact PPI interaction site were detected. There was minimal to no enrichment over background for I867A and Δ10. A Flag ChIP-qPCR for MLL-AF9 showed no significant difference in localization to both HoxA9 and Meis1 promoters in each cell line. **(B)** With an antibody for H3K79me2, we confirmed the decrease in methylation at the Meis1 gene locus t intron 8 in the MigR1, Δ 10, I867A and RCR cells in comparison to WT with corresponding % input control. (representative data from 2 independent experiments) .

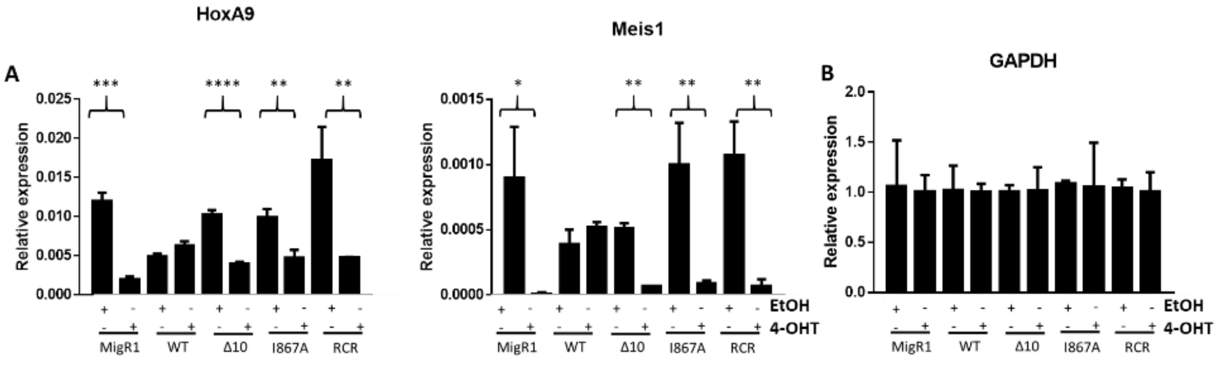

Figure S5. Raw qPCR data for *HoxA9*/*Meis1* gene expression. (A) Raw data used for the main figure showing the decrease in HoxA9 and Meis1 after 4-OHT treatment in the mutant and empty vector cell lines and not in WT cells. **(B)** GAPDH control.

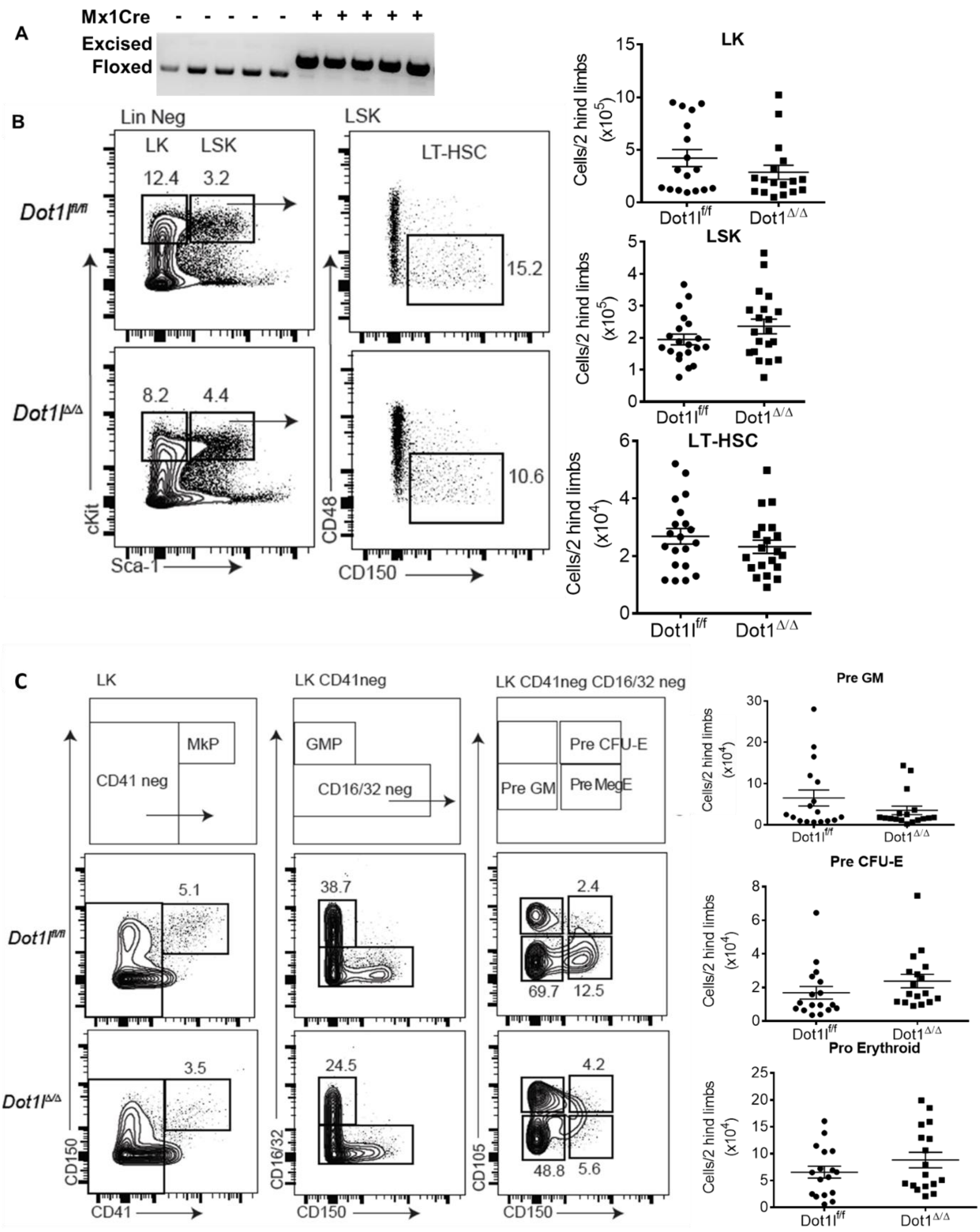

Figure S6. Day 7 of *Dot1l* inactivation shows early impacts some progenitor cell populations. (**A**) Excision PCR showing high excision efficiency in *Dot1l$^{f/f}$ Mx-Cre*+ mice (n=5/group, representative of 5 independent experiments). **(B)** Flow cytometric analysis of LSK and LT-HSCs (CD150^{+}CD48^{-}LSK), showing no alterations in Dot1l-null HSCs 7 days post deletion (n=20/group; pooled from 5 independent experiments; mean +/- SEM). Quantification of LK, LSK and LT-HSC. **(C)** Flow cytometric analysis of progenitor cell compartments. MkP (CD41^{+}CD150^{+}LK), GMP (CD16/32^{+}CD150^{-}CD41^{-}LK), Pre CFU-E (CD150^{+}CD105^{+}CD16/32^{-}CD41^{-}LK), Pre MegE (CD150^{+}CD105^{-}CD16/32^{-}CD41^{-}LK), Pre GM progenitors (CD150^{-}CD105^{-}CD16/32^{-}CD41^{-}LK). Quantification shows a decrease in Pre-GM cells in mice lacking *Dot1l* and no changes in Pre CFU-E and Pro-Erythroid cells. (n=17/group; pooled from 5 independent experiments; mean +/- SEM).

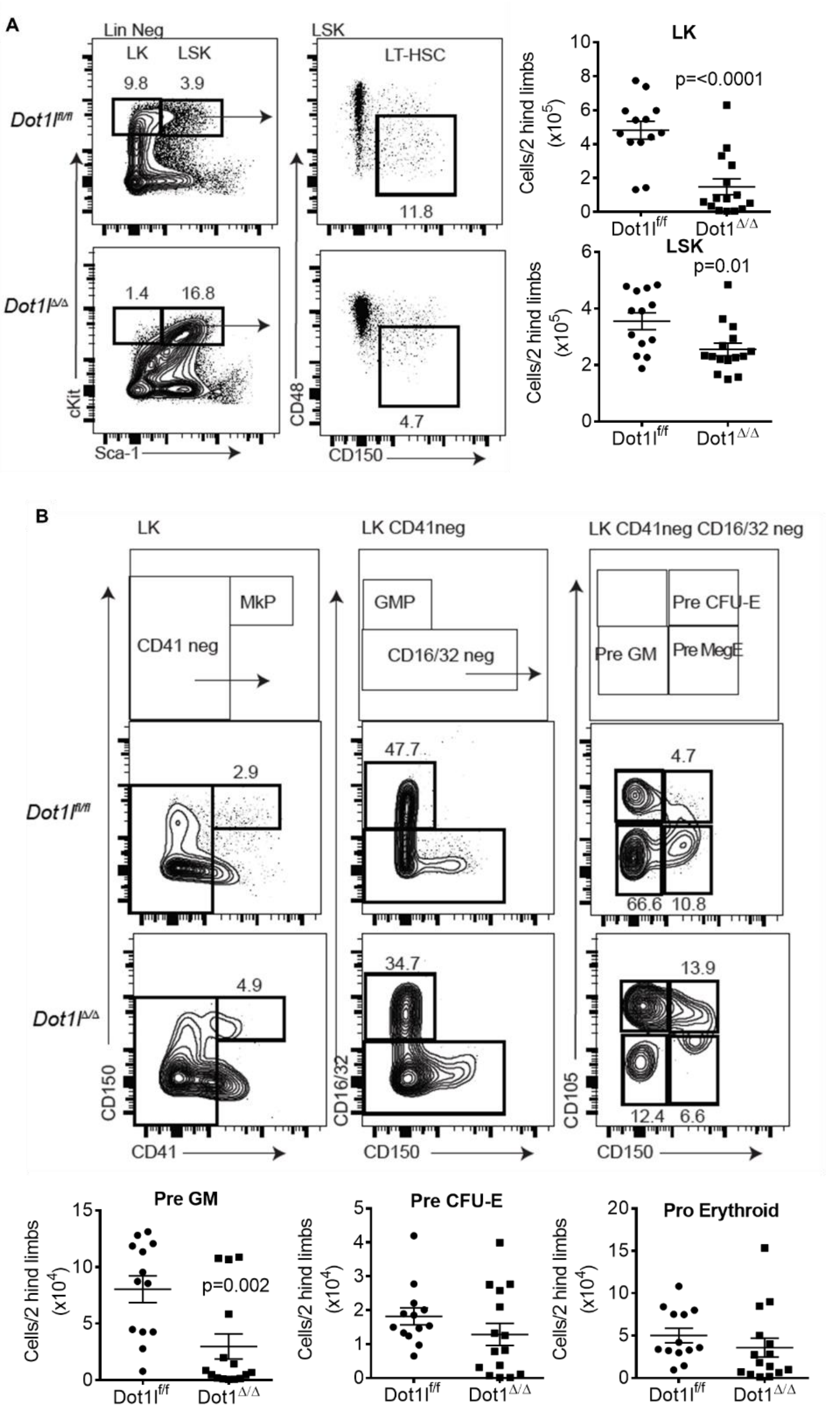

Figure S7. HSCs and progenitors are depleted 10 days after *Dot1l* inactivation**. (A)** Flow cytometric analysis of LK, LSK and LT-HSCs ($CD150^+CD48^-LSK$), showing a decrease in *Dot1l-null* HSCs 10 days post deletion. (n=13-15/group; pooled from 3 experiments; mean +/- SEM). Quantification of LK and LSK. **(B)** Flow cytometric analysis of progenitor cell compartments. MkP ($CD41^+CD150^+LK$), GMP ($CD16/32^+CD150^-CD41^-LK$), Pre CFU-E ($CD150^+CD105^+CD16/32^-CD41^-LK$), Pre MegE ($CD150^+CD105^-CD16/32^-CD41^-LK$), Pre GM progenitors ($CD150^-CD105^-CD16/32^-CD41^-LK$). Loss of *Dot1l* decreases Pre-GM, Pre CFU-E and Pro Erythroid progenitors. (n=13-15/group; pooled from 3 experiments; mean +/- SEM).

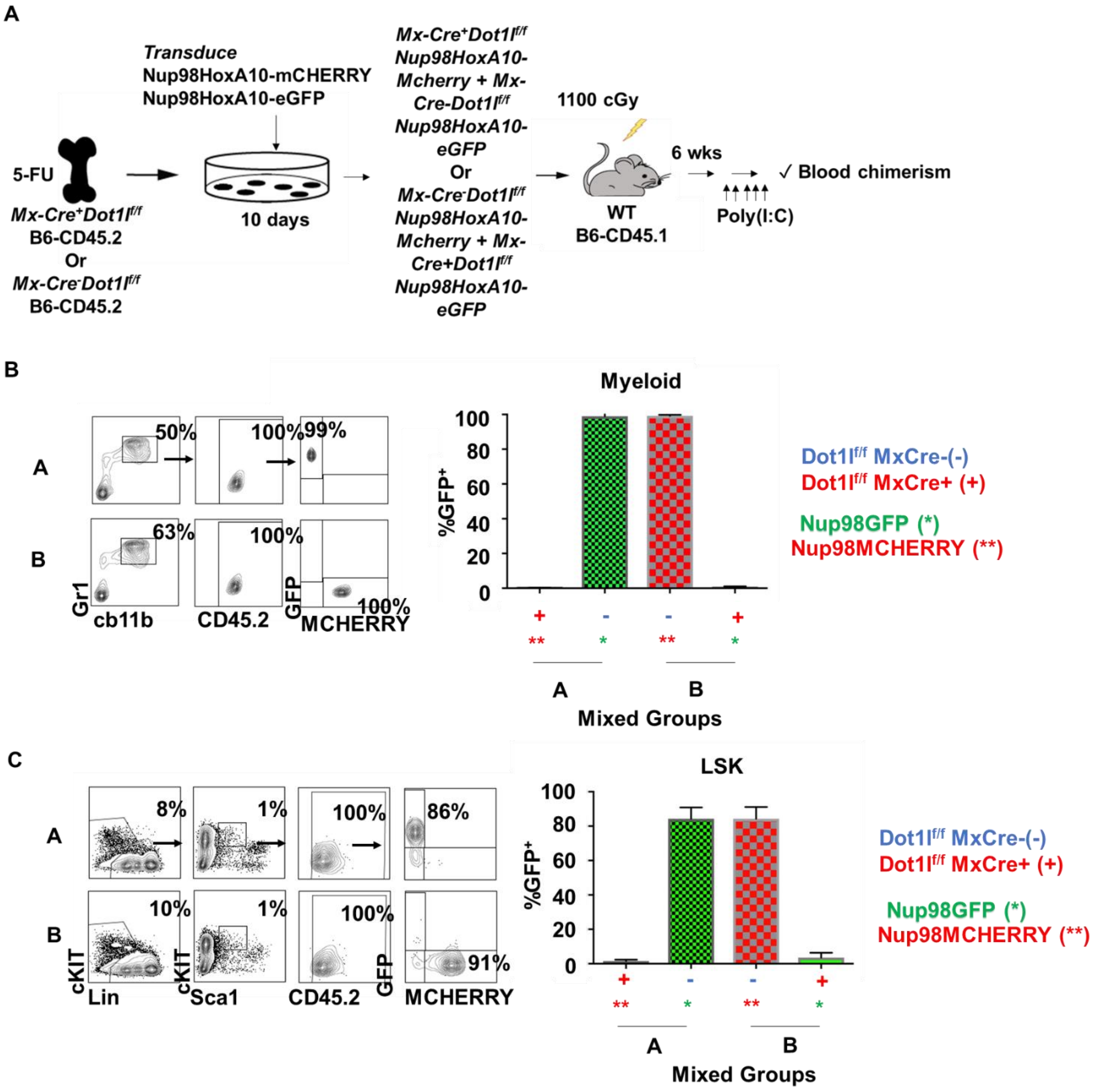

Figure S8. Nup98HoxA10 does not rescue DOT1L loss**. (A)** Experimental approach of Nup98HoxA10 control experiment. Bone marrow from *Mx1-Cre+ Dot1f/f* or *Mx1-Cre- Dot1f/f* B6-CD45.2 was transduced with *Nup98HoxA10*-mCherry and *Nup98HoxA10*-eGFP. The cells were mixed so that Cre+ and Cre- cells had different Nup98HoxA10 colors to follow their growth *in vivo*. Cell mixtures were transplanted into WT B6-CD45. 1 mice and 5 injections of poly(I:C) was administered 6 weeks post-transplant. **(B)** (left)Representative flow panels showing that the myeloid population is only comprised of *Mx1-Cre- Dot1f/f Nup98HoxA10*; whereas, *Mx1-Cre+ Dot1f/f* B6-CD45.2 *Nup98HoxA10* cells are not able to grow *in vivo*. (right) Quantification of all flow data. **(C)** (left)Representative flow panels showing the LSK population is only comprised of *Mx1-Cre- Dot1f/f Nup98HoxA10*; whereas, *Mx1-Cre+ Dot1f/f* B6-CD45.2 *Nup98HoxA10* cells are not able to grow *in vivo*. (right) Quantification of all flow data.

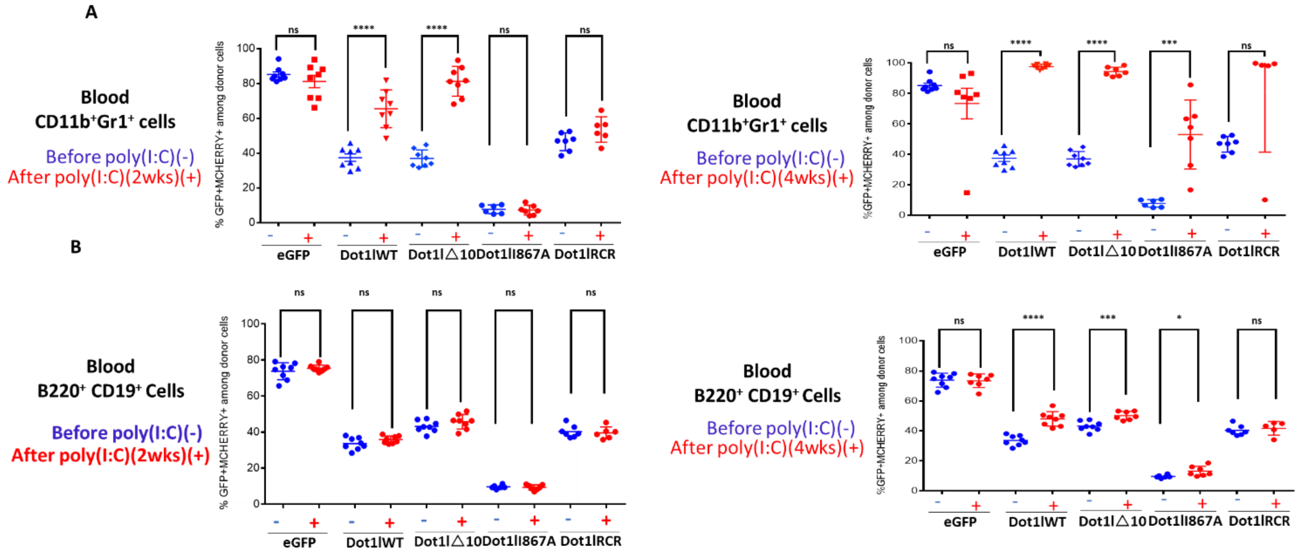

Figure S9. The AF9-binding domain of DOT1L is dispensable for adult hematopoiesis. **(A)** Quantification of flow cytometric data showing that donor *Mx1-Cre+ Dot1f/f Nup98HoxA10* DOT1L WT and DOT1L Δ10 cells sustain the myeloid compartment in host mice 2 weeks (left) and 4 weeks (right) after poly(I:C) injection. DOT1L I867A cells maintained low initial chimerism at 2 weeks, possibly due to a low transduction rate, but showed subsequent selection for transduced cells at 4 weeks. In contrast, *Mx1-Cre+ Dot1f/f Nup98HoxA10* transduced with eGFP only or with DOT1L RCR (lacking DOT1L enzymatic activity) showed no significant changes in chimerism at 2 and 4 weeks, consistent with the absence of selective advantage for transduced cells. **(B)** Similar data focused on chimerism in the B220+ CD19+ B cell compartment at 2 and 4 weeks post poly(I:C) injection.

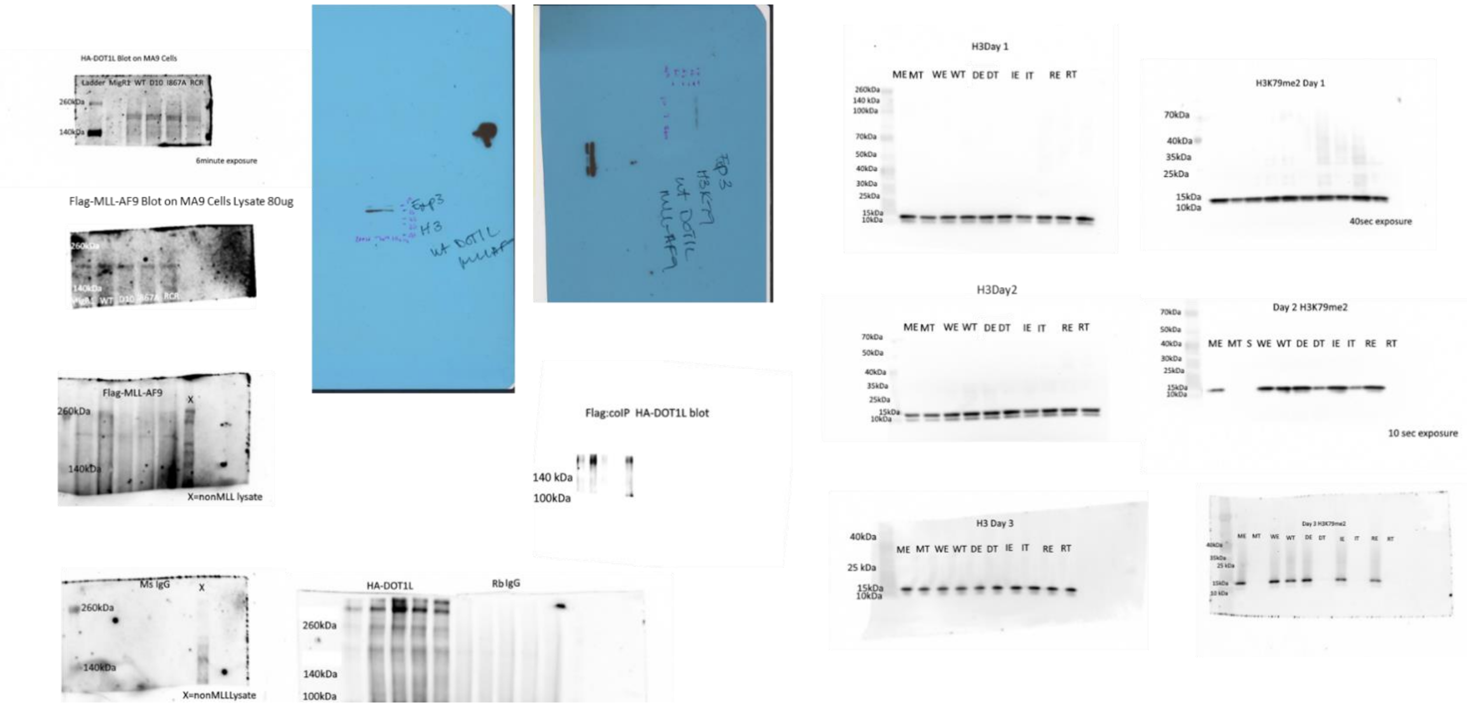

Figure S10. Original Western Blots.

References:

1. 1. Jo SY, Granowicz EM, Maillard I, Thomas D, Hess JL. Requirement for Dot1l in murine postnatal hematopoiesis and leukemogenesis by MLL translocation. *Blood*. 2011;117(18):4759-4768.
